# Study of Energy Saving Using Silica Aerogel Insulation in a Residential Building

**DOI:** 10.3390/gels9020086

**Published:** 2023-01-19

**Authors:** Conal Thie, Sean Quallen, Ahmed Ibrahim, Tao Xing, Brian Johnson

**Affiliations:** 1 Department of Mechanical Engineering, University of Idaho, Moscow, ID 83844-0902, USA; 2 Department of Civil & Environmental Engineering, University of Idaho, Moscow, ID 83844-1022, USA; 3 Schweitzer Engineering Laboratories Endowed Chair in Power Engineering, Distinguished Professor, Department of Electrical and Computer Engineering, University of Idaho, Moscow, ID 83844-1023, USA

**Keywords:** aerogel, insulation, simulation

## Abstract

Energy consumption, specifically in the building sector, is expected to rise. One potential way to reduce energy consumption, or to slow this increase, is to reduce the heat loss in residential homes. Silica aerogels have grown in popularity as an insulating material due to their extremely low thermal conductivity. However, the benefits of using silica aerogels as an insulator in residential buildings have not been thoroughly studied. To understand the benefits of using silica aerogels as a thermal insulator in residential homes, experimentally validated simulations were performed. The simulations were performed on a model of a full-scale residential house using the multiphysics software ANSYS FLUENT 2019 R2. The simulations helped predict the actual saving benefits of using aerogels as an insulator. Aerogels have the potential to be used as an insulator in both the walls and windows due to its semitransparency. The results showed that the average kWh savings using one half-inch layer of wall aerogel insulation coupled with window aerogel insulation was 20.9% for the single-family house compared to traditional insulation. On average, the energy lost through the windows was 39.1% lower when using aerogel insulation compared to standard insulating materials. The energy lost through the house walls was 13.3% lower on average when using a thin layer of aerogel insulation. While a thin layer of aerogel insulation provided a benefit when used in the house walls, the potential for savings per quantity used was greater in the windows.

## 1. Introduction 

Global energy and environmental issues call for an urgent reduction in energy consumption and greenhouse gas emissions. Over the last decade, about 40% of the total U.S. energy use was consumed in residential and commercial buildings [1]. It is estimated that the global energy demand in buildings will at least be doubled by 2050 compared to today’s levels [2]. As economic growth and urbanization are expected to continue, the energy consumption in the building sector will keep growing. Slowing the growth of energy consumption in the building sector will serve to reduce ownership costs and to slow the increase in greenhouse gas emissions. The installation of thermal insulation is one of the most effective approaches to improving the energy efficiency of buildings [1,3,4,5]. There are two methods of improving this thermal insulation; the first is to continue using traditional building insulators, such as mineral wool [6,7], and to increase the total amount of insulation. The drawback of increasing the insulation used is a reduction in floor space and, eventually, an increase in cost. Research results indicated that mineral wool systems showed a more negative environmental impact when considering all the environmental indicators rather than the equivalent systems with expanded polystyrene [8]. The second method of improving thermal insulation is to improve the existing insulation systems or to develop new materials to use as insulators. This is the key tool in designing and constructing energy-efficient buildings. For example, the IEA-EBC Annex 65 Project [9] aims to evaluate the long-term performance of superinsulating materials in building components and systems with a focus on two superinsulating materials, i.e., vacuum insulation panels and advanced porous materials [10,11].

New state-of-the-art insulators [6] include vacuum insulation panels [4,12], gas-filled panels [13], aerogels [14,15,16], and thermal insulation materials [17,18]. One of the most promising new insulators is silica aerogels [19]. The primary reason aerogels are so appealing is their very low thermal conductivity [20]. Commercial aerogels have a thermal conductivity that is typically in the range of 0.013–0.015 W/(m·K). In comparison, mineral wool typically has a thermal conductivity between 0.03 and 0.04 W/(m·K). Another characteristic that makes aerogels standout compared to other insulators is their potential transparency [20]. This opens the possibility of aerogels being used as a window and skylight material, not just wall insulation.

A comprehensive review of aerogel thermal insulation cementitious composites was introduced by the authors of [21]. The study presented an in-depth review of the production, mechanical, and durability properties of aerogel composite insulation. A summary of case studies was presented with a strong suggestion for the future implementation of such materials in building insulation. Aerogel insulation implementation has spread to various countries under various climactic environments. For example, the performance of aerogels implemented as a thermal insulation material in residential buildings was evaluated under tropical climates in Nigeria [22]. The study showed a 15% reduction in energy consumption and stated that it was a great potential investment for the energy sector. The authors expressed concern that the high initial price of aerogels might limit their usage. Ganobjak et al. [23] developed a novel insulation system using silica aerogel granules. The authors tested the thermal and mechanical properties, and the results were compared to 3D computer simulations of glass–brick wall systems. The thermal conductivity of the glass–brick system was 53 mW/(m·K), and it matched the computer modeling well. The authors claimed that the developed system was one of the insulation systems that had the highest performance available in the literature [23].

The development of aerogel insulation using aramid fiber composites was introduced in [24]. The study included the synthesis of aerogels using tetraethoxysilane (TEOS) as the precursor, polyimide (PI) powder as the reinforcing agent, and nonwoven AF as the substrate. The developed insulation material was characterized using scanning electron microscopy (SEM) accompanied by mechanical testing. The results demonstrated that the aerogel insulation showed an excellent heat transfer performance and that the thermal conductivity decreased from 4.08 to 3.91 (W/cm·°C) × 10^−4^. Carroll et al. [25] presented various approaches for preparing monolithic silica aerogel windows using a supercritical extraction method. The results presented a glazing design that used thinner monoliths incorporated with artistic dyes and laser etching.

Aerogels as an alternative for thermal insulation in buildings were investigated in [26] through an in-depth review. The review included a comprehensive description of the most relevant properties of aerogels and their insulation capabilities. The effect of silica aerogels on thermal insulation and the acoustic absorption properties of geopolymer foam composites were presented in [27]. The study included four types of silica aerogels as potential energy-saving materials. The aerogels had several ranges of particle sizes (2–40 μm, 100–700 μm, 100–1200 μm, and 700–4000 μm) in order to investigate their insulation and acoustic properties. The results showed that the smaller the aerogel particle size, the less effective it was as insulation. The optimum aerogel type showed a thermal conductivity of 0.133 W/(m·K). Yin et al. [28] investigated the thermal performance of an enclosed dome with a double-layered aerogel–glass insulation system. The investigation was conducted experimentally using thermometers to monitor the temperature during the summertime. The study parameters were the thermal insulation options: a double-layered membrane roof containing no extra insulation, peripheral wool glass insulation, and all aerogel insulation. The roof with hybrid insulation reduced the average temperature difference by 7.7 °C. Finally, the thermal insulation and moisture resistance of the high-performance silicon aerogel composite foam ceramic and foam glass were investigated in [29]. The authors synthesized new ceramic composite aerogel and foam glass composite aerogel materials. The thermal conductivities were 0.04159 and 0.04424 W/(m·K) at 25 °C for the aerogels and the foam glass composites, respectively. The developed materials showed great potential as insulation for building structures.

While using aerogels is expected to significantly decrease the annual heat loss in residential buildings compared to standard insulation materials [14], the literature contains very limited research conducted on the implementation of aerogel insulation in walls and windows. In addition, there are few full-scale experiments or simulations conducted showing the true potential savings of using aerogels [30,31,32]. The objectives of this study were to determine the thermal properties of silica-aerogel-based walls and windows, to validate these values experimentally, and to apply these validated values to high-fidelity multiphysics simulations. The annual heat loss in a residential building with aerogel insulation was compared to the same building with standard insulation. By completing these objectives, the main contribution of this study was determining the potential energy savings of using aerogels as an insulator in a residential house either as a retrofit to an existing house or in a new house construction. Compared with the previous literature, this study excluded other factors affecting the energy consumption of the residential buildings, such as human activities, and, therefore, facilitated the evaluation of the energy savings as a result of a reduction in the thermal conductivity of the silica aerogels only.

## 2. Results and Discussion

The simulation was run as a parametric study. The proposed parametric study factors were whether aerogels were used in the walls or whether aerogels were used in the windows. Therefore, there was a total of four simulation runs as shown in Table 1. This included the control simulation that did not have aerogels in either the windows or the walls. These studies were performed to see the practical effect of using aerogels in either the walls or the windows and to determine if one was more useful than the other by comparing the results to the control simulation that did not include any aerogels. The amount of the energy savings for each case was reported in the results.

The temperature data taken from The National Centers for Environmental Information [33] were averaged for each month as shown in Table 2. Each simulation scenario in the parametric study was solved for each month of the year. The temperature values in Table 2 were used as the outer-wall temperatures for each month. This approach was taken to reduce the computational costs.

**Figure 1:**
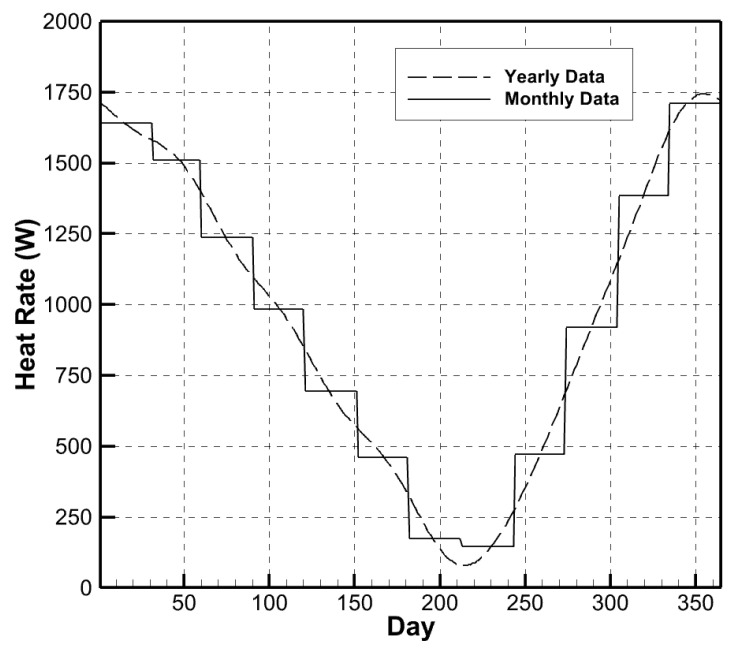
Comparison of wall heat rates using yearly and monthly data.

To authenticate this approach, one simulation was conducted to calculate the wall heat rates using the average temperature values for each day. This 365-day simulation was performed on a residential house with standard insulation, i.e., no aerogel insulation. This simulation was compared to the control simulation using the monthly data (Figure 1).

**Figure 2:**
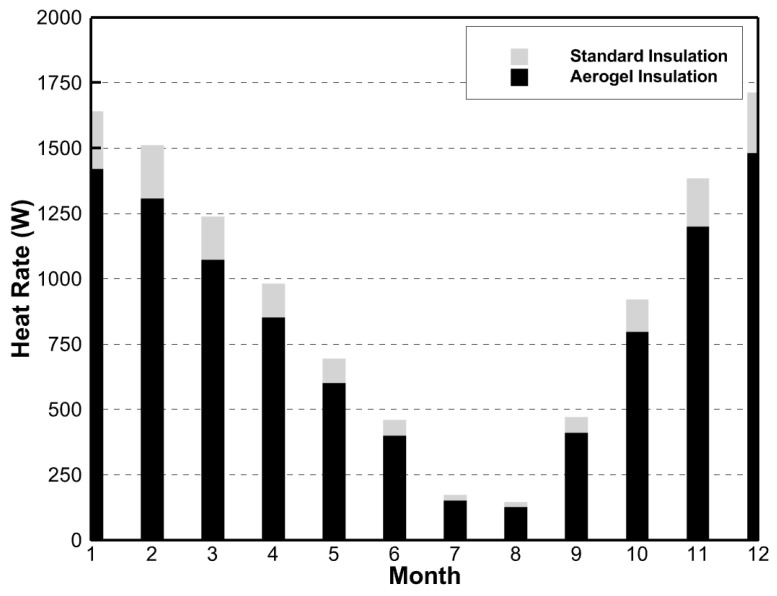
Simulation results of rate of heat lost through house walls comparing aerogel to standard insulation.

**Figure 3:**
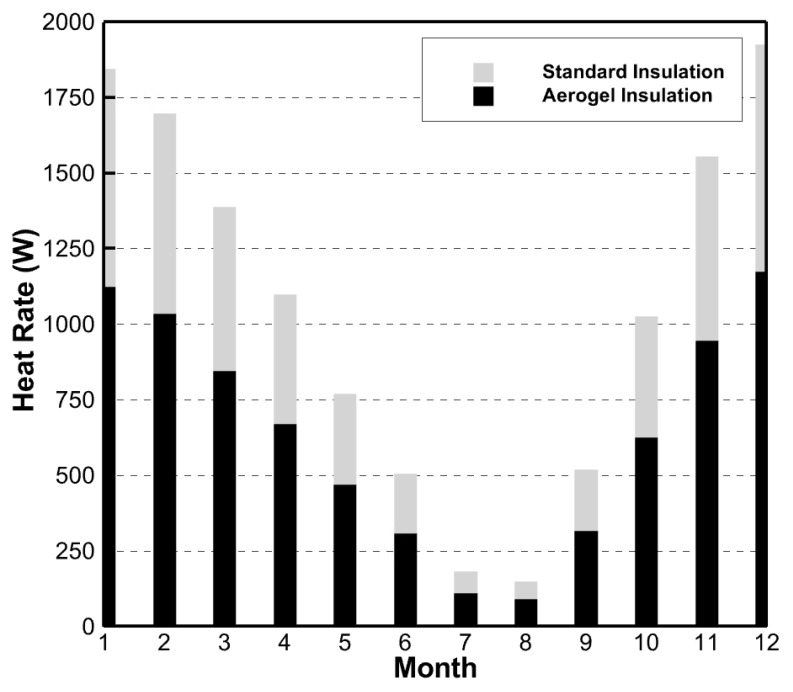
Simulation results of rate of heat lost through house windows comparing aerogel to standard insulation.

The rate of the heat lost through the walls and windows was solved in the simulations. Figure 2 and Figure 3 show these heat rates when aerogels were used versus standard insulation.

From Figure 2, the average monthly heat lost through the house walls was 13.3% lower when using a ½ inch layer of aerogels rather than standard insulation. Likewise, Figure 3 shows that the average monthly rate of heat lost through the house windows was 39.1% lower when using aerogels in the window gap. It is clear from these results that, when used as an insulator, aerogels reduced the rate of the heat lost through the house’s walls and windows. It is important to note that the heat loss rate difference between aerogel insulation and standard insulation was much greater in the windows than in the walls.

**Figure 4:**
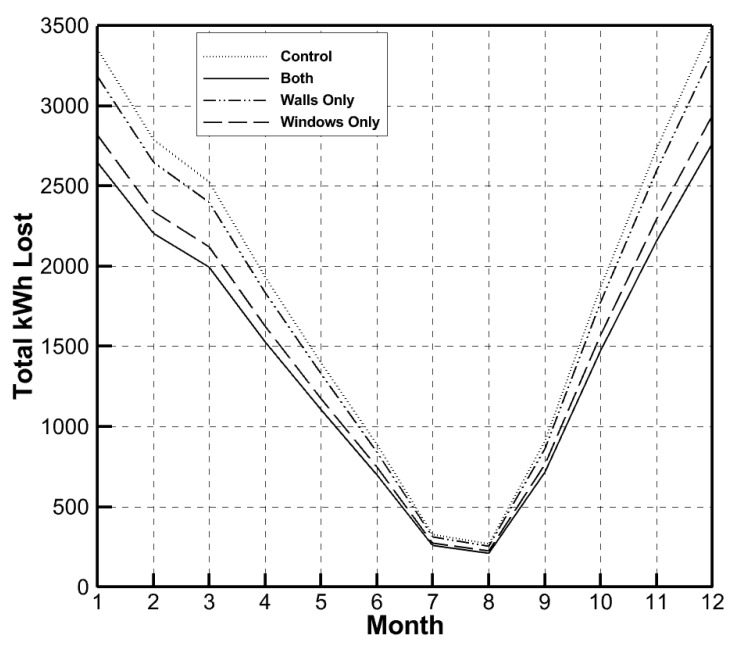
Total kWh loss per month for each simulation scenario.

The total kWh per month was calculated for each of the four simulations with the following equation:

(1) $$E=24H_{r}D\;\;$$

where *H_r_* is the total rate of the heat lost through the house for each month (kW) and where *D* is the number of days in the month. Figure 4 shows this kWh loss per month for each simulation.

The kWh loss in Figure 4 represents the energy loss due to the heat lost throughout the entire house, including the windows, walls, floor, doors, etc. However, it does not account for many forms of energy loss in residential homes, such as the appliances, opened windows, opened doors, etc. As expected, the greatest energy loss occurs in the months of November, December, January, and February. The average kWh used over the entire year for the house when no aerogels were used (control) was 1874 kWh, and, when aerogels were used in both the windows and walls (both), the average kWh was 1480 kWh. Thus, the average kWh per month was 20.9% lower when using aerogels as an insulator. It is clear from the results that the lowest loss of energy occurred when the residential house had aerogels in both the windows and walls. Despite there being more aerogels used when placed in the walls than when placed in the windows, the benefit from using aerogels in the windows was greater than that of its use in the walls.

## 3. Conclusions from Simulation Study

The feasibility of using aerogel blankets in the walls and windows as a super insulator was verified using small-scale laboratory testing and high-fidelity computer simulations. The following conclusions were drawn from the project tasks conducted:

- The multiphysics model was confirmed to be a promising tool for predicting room-temperature decay.
- Multiphysics simulations can be used to accurately predict the temperature, heat flux, and energy loss through the windows, walls, floors, doors, ceilings, and their combination under various insulation conditions that are difficult to create using experiments.
- The energy lost through the walls of a house is potentially 13.3% lower when using a ½ inch layer of aerogels as insulation.
- The energy lost through the windows of a house is potentially 39.1% lower when using a ½ inch layer of aerogels as insulation.
- The potential yearly energy savings using a ½ inch layer of aerogel insulation in the window gaps and walls of a residential building is 4721.8 kWh. This is more than one-fifth the total cost without aerogel insulation.
- The savings potential is greater with the use of aerogel insulation in the windows than in the walls.
- Future work will focus on adding other factors, such as human activities, into the simulations.

## 4. Materials and Methods

The primary multiphysics software used to perform the simulations was the ANSYS FLUENT 2019 R2 suite [34]. FLUENT is a multiphysics software that can perform many functions, including computational fluid dynamics coupled with heat transfer. Other software was used alongside the ANSYS software, including Tecplot 360 EX 2019 R1 for postprocessing and Pointwise software for meshing.

### 4.1. Mathematical Modeling

The governing heat transfer equation used in FLUENT was the equation for conduction or the differential form of Fourier’s law.

(2) q=−k∇T  

where ***q*** is the local heat flux density (W/m^2^), k is the thermal conductivity of the material (W/(m·K)), and ∇T is the temperature gradient (K/m).

### 4.2. Validation

**Figure 5:**
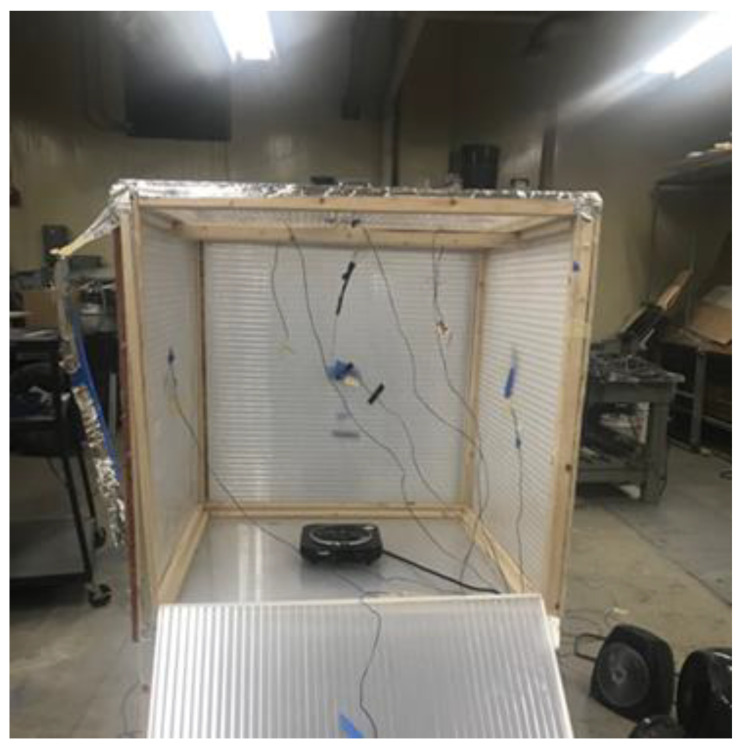
Aerogel box for experimental validation.

To validate the ANSYS simulations, two experiments were performed using two different aerogels. The first aerogel was a blanket type and was meant to mimic house wall insulation. Its commercial name was Spaceloft^®^ blanket aerogel, and it was purchased from Aspen Aerogels (Northborough, MA, USA). The other aerogel type was a semitransparent glazed aerogel meant to mimic aerogel windows [35]. This was Lumira^®^ aerogel purchased from Duo-Gard Industries Inc. (Canton, MI, USA). The two experiments were set up in a similar setup and were under the same environmental conditions (temperature and relative humidity). Two 3.0 × 3.0 × 3.0 foot cubical wooden frames were constructed as shown in Figure 5. One set up was made with aerogel blankets as wall insulation (Spaceloft^®^), and other was made with walls constructed with glazed aerogel windowpanes (Lumira^®^). Figure 5 shows the setup using the Lumira^®^ aerogel window material with one side lowered to display the inside of the box.

**Figure 6:**
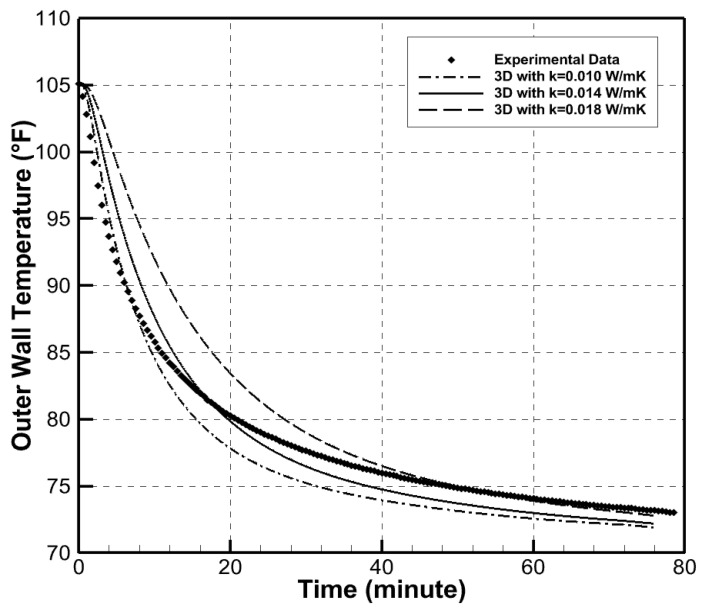
Comparison of simulation results to experimental results of Spaceloft^®^ aerogel wall material.

A heater was placed inside along with temperature sensors on the inside and outside of each wall. The temperature gradient between the inside and outside of the box were measured, and all data were collected using a datalogger. The main purpose of this box was to measure the thermal conductivity of the aerogels and to validate the value provided by the manufacturer. Figure 6 shows the three simulation temperature gradients compared to the experimental temperature gradient. The thermal conductivity value that matched the experimental results was *k* = 0.014 W/(m·K).

As shown in Figure 6, the experimental and the simulation temperature-gradient curves matched very well. Thus, the k-value used in the multiphysics simulations was validated through the experimental procedure.

**Figure 7:**
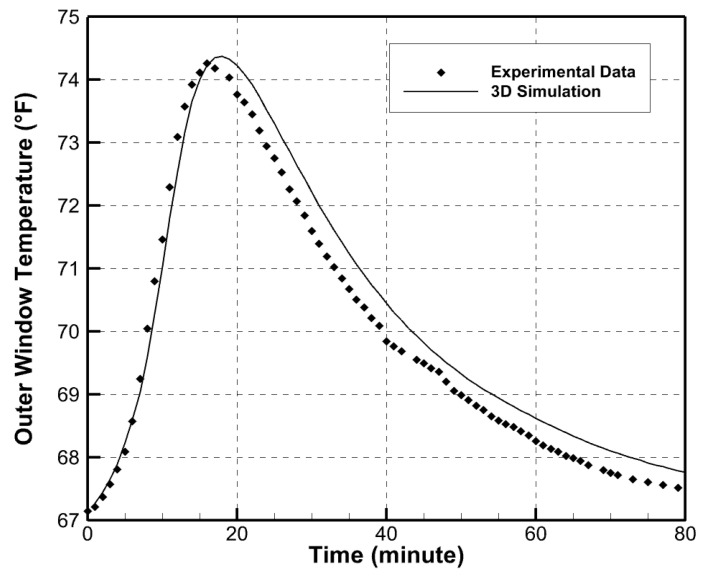
Comparison of simulation results to experimental results of Lumira^®^ aerogel window material.

A replicate experiment was performed using the Lumira^®^ aerogel window material. The previously validated simulation tool was used to simulate this experiment, and, again, the prediction agreed well with the experimental data as shown in Figure 7.

### 4.3. Simulation of a Residential House

To understand the effects of aerogels as an insulator, a steady-state simulation of a full-scale 3D residential house was performed. The insulation of the thickness of the house’s walls and window was modeled as variable. This enabled a parametric study to be performed, using both standard insulation and aerogel insulation. Outside temperatures in all the simulations were based on the measured daily temperature averages of Moscow, Idaho, over the dates 1 January 2015–31 December 2015, which were taken from the National Centers for Environmental Information (NCEI) [33]. The NCEI provide environmental and climate data for many purposes, including academia. The year was chosen due to the large amount of data available for it. The flux across the walls and windows were simulated, and the impact of the aerogels on the overall thermal environment was evaluated. The total heat loss during each month was evaluated in units of kWh. The house design was based off the floor plans for a residential home in Moscow, Idaho. The design was simplified and modified to reduce computational costs, but the important features were kept. Some of the features that were simplified include the attic, roof, and basement.

#### 4.3.1. Geometry and Grid Development

**Figure 8:**
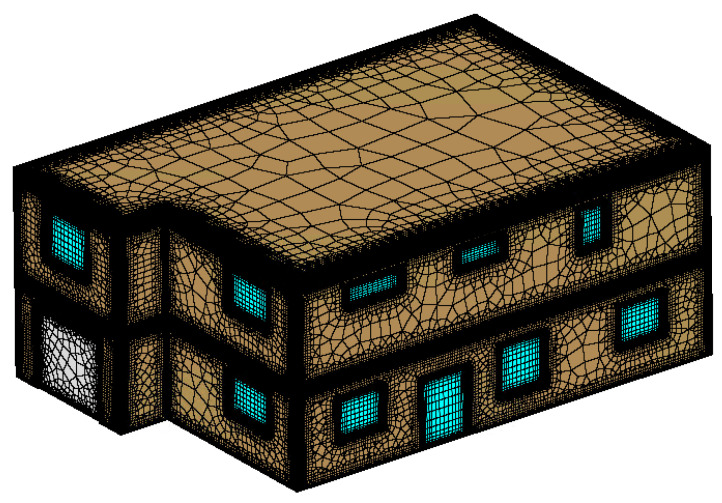
Residential house CFD mesh.

A grid of the single-family house was developed for multiphysics simulation to evaluate heat loss for a typical residential house with the addition of aerogel windows and walls. Figure 8 shows the 3D view of the mesh of the single-family house used in the computational fluid dynamics (CFD) simulations. This mesh was generated in Pointwise.

A multilayer floor and external walls were modeled. The house had a floor area plan of 2000 square feet. Since the focus was on walls and windows, a flat ceiling was used to reduce unneeded simulation complexities. Other features included multiple internal walls, windows, and doors. The single-family house grid contained 11.4 million grid points. To compare the effects of aerogels, the vertical walls and windows had variable materials. The wall insulation was 4 inches thick, including a ½ inch variable section that could be specified as either an aerogel or generic insulation material composite, with a thermal conductivity value of *k* = 0.045 W/(m·K) [6]. The walls were composed of outer plywood sheathing with ½ inch rated material, leaving the walls with 5.5 inch of effective insulation (R-21). The walls were broken into two blocks (a 5 inch block and a 1/2 inch block). Studs were 16 inch in center with 1/2 inch gypsum wallboard on the walls’ interior. The windowpanes had a gap (½ inch) that could be specified as either air or aerogel insulation. For this case, the thermal conductivity of air used was *k* = 0.0242 W/(m·K). This allowed for the ability to isolate and swap different window/wall configurations in the main grid. The house grid included a total of 20 windows varying in size, 2 sliding glass doors, 1 garage door, and 1 standard door. The ground floor was 4 inch concrete with 60°F ground outside of it. There was no basement, so the cement sat directly on the ground underneath. Sheathing was applied to outside walls. Other materials were used throughout the house as well to generate a realistic simulation. Table 3 includes all the materials used as well as their thermal properties as used in the simulation.

#### 4.3.2. Solver Methods

FLUENT solver 19.4 was used. The FLUENT model settings chosen are seen in Table 4.

The momentum equation was solved, although it was not necessary in this case since there was no fluid flow. The termination criterion under the multigrid method for all the equations was set to 0.1, and the energy relaxation factor was set to 1. The minimum and maximum absolute pressure limits were 1 Pa and 5 × 10^10^ Pa, respectively, while the minimum and maximum temperature limits were 1 °C and 5 × 10^3^ °C, respectively.

#### 4.3.3. Boundary Conditions

The primary thermal boundary conditions that were controlled in the parametric study were as follows: floor, inner wall, outer wall, variable wall layer, ceiling, and window gap. The inner-wall boundary condition was set at a constant room temperature of 70°F, while the outer-wall temperature was set as the average outside temperature for each month.

## Figures and Tables

**Table 1:** Parametric study.

Study Type	Aerogels in Window	Aerogels in Walls
Control	No	No
Walls Only	No	Yes
Windows Only	Yes	No
Both	Yes	Yes

**Table 2:** Averaged monthly temperatures, Moscow Idaho, 2015.

Month	January	February	March	April	May	June	July	August	September	October	November	December
Mean Temperature (°F)	31.2	34.3	40.8	46.9	53.8	59.4	66.2	66.9	59.1	48.4	37.3	29.5

**Table 3:** Simulation materials.

Material	Location	Density kg/m^3^	Specific Heat J/(kg·K)	Thermal Conductivity W/(m·K)
Wood	Walls and roof	700	2310	0.173
Vinyl	Siding	125	1200	0.17
Sheathing	Roof	6	1300	0.055
Insulation Composite	Walls	73.1875	1062.8	0.045
Gypsum	Walls	800	1100	0.17
Glass	Windows	2500	800	0.8
Concrete	Foundation	2300	880	1.4
Aerogel	Windows and Walls	120	1050	0.014
Air	N/A	1.225	1006	0.0242

**Table 4:** FLUENT model settings.

Model	Setting
Space	3D
Time	Steady
Viscous	Laminar
Heat Transfer	Enabled
Solidification and Melting	Disabled
Radiation	None
Species	Disabled
Coupled Dispersed Phase	Disabled
NO_x_ Pollutants	Disabled
SO_x_ Pollutants	Disabled
Soot	Disabled
Mercury Pollutants	Disabled

## Data Availability

All the data generated in this research have been included in this paper.

## References

[1] Cao X., Dai X., Liu J. (2016). Building energy-consumption status worldwide and the state-of-the-art technologies for zero-energy buildings during the past decade. Energy Build..

[2] Berardi U. (2017). A cross-country comparison of the building energy consumptions and their trends. Resour. Conserv. Recycl..

[3] Pérez-Lombard L., Ortiz J., Pout C. (2008). A review on buildings energy consumption information. Energy Build..

[4] Fantucci S., Garbaccio S., Lorenzati A., Perino M. (2019). Thermo-economic analysis of building energy retrofits using VIP-Vacuum Insulation Panels. Energy Build..

[5] Sun Y., Wilson R., Wu Y. (2018). A Review of Transparent Insulation Material (TIM) for building energy saving and daylight comfort. Appl. Energy.

[6] GhaffarianHoseini A., Dahlan N.D., Berardi U., GhaffarianHoseini A., Makaremi N., GhaffarianHoseini M. (2013). Sustainable energy performances of green buildings: A review of current theories, implementations and challenges. Renew. Sustain. Energy Rev..

[7] Lyons A. (2014). Materials for Architects and Builders.

[8] Michalak J., Czernik S., Marcinek M., Michałowski B. (2020). Environmental burdens of external thermal insulation systems. expanded polystyrene vs. mineral wool: Case study from Poland. Sustainability.

[9] VIPA International IEA-EBC Annex 65 Project. https://vipa-international.org/iea-ebc-annex-65-project/.

[10] Kucukpinar E., Miesbauer O., Carmi Y., Fricke M., Gullberg L., Erkey C., Caps R., Rochefort M., Moreno A.G., Delgado C. (2015). Development of transparent and opaque vacuum insulation panels for energy efficient buildings. Energy Procedia.

[11] Lu X., Caps R., Fricke J., Alviso C., Pekala R. (1995). Correlation between structure and thermal conductivity of organic aerogels. J. Non-Cryst. Solids.

[12] Uriarte A., Garai I., Ferdinando A., Erkoreka A., Nicolas O., Barreiro E. (2019). Vacuum insulation panels in construction solutions for energy efficient retrofitting of buildings. Two case studies in Spain and Sweden. Energy Build..

[13] Jukić M., Jordan S., Lisičić D. (2019). Novel thermal insulation with gas-filled cavities–assessment of thermal performance of different designs based on numerical simulations of heat transfer. Int. J. Comput. Methods Exp. Meas..

[14] Jelle B.P., Baetens R., Gustavsen A. (2015). Aerogel insulation for building applications. Sol-Gel. Handb..

[15] Ramesh M., Rajeshkumar L., Balaji D. (2021). Aerogels for insulation applications. Mater. Res. Found..

[16] Wang L., Feng J., Luo Y., Zhou Z., Jiang Y., Luo X., Xu L., Li L., Feng J. (2021). Three-Dimensional-Printed Silica Aerogels for Thermal Insulation by Directly Writing Temperature-Induced Solidifiable Inks. ACS Appl. Mater. Interfaces.

[17] da Cunha S.R.L., de Aguiar J.L.B. (2020). Phase change materials and energy efficiency of buildings: A review of knowledge. J. Energy Storage.

[18] Al-Yasiri Q., Szabó M. (2021). Incorporation of phase change materials into building envelope for thermal comfort and energy saving: A comprehensive analysis. J. Build. Eng..

[19] Shah S.N., Mo K.H., Yap S.P., Radwan M.K. (2021). Towards an energy efficient cement composite incorporating silica aerogel: A state of the art review. J. Build. Eng..

[20] Baetens R., Jelle B.P., Gustavsen A. (2011). Aerogel insulation for building applications: A state-of-the-art review. Energy Build..

[21] Adhikary S.K., Ashish D.K., Rudžionis Ž. (2021). Aerogel based thermal insulating cementitious composites: A review. Energy Build..

[22] Bashir A.W., Leite B.C.C. (2022). Performance of aerogel as a thermal insulation material towards a sustainable design of residential buildings for tropical climates in Nigeria. Energy Built Environ..

[23] Ganobjak M., Malfait W.J., Just J., Käppeli M., Mancebo F., Brunner S., Wernery J. (2022). Get the light & keep the warmth-A highly insulating, translucent aerogel glass brick for building envelopes. J. Build. Eng..

[24] Xue R., Liu G., Liu F. (2023). A simple and efficient method for the preparation of SiO2/PI/AF aerogel composite fabrics and their thermal insulation performance. Ceram. Int..

[25] Carroll M.K., Anderson A.M., Mangu S.T., Hajjaj Z., Capron M. (2022). Aesthetic Aerogel Window Design for Sustainable Buildings. Sustainability.

[26] Balaji D., Sivalingam S., Bhuvaneswari V., Amarnath V., Adithya J., Balavignesh V. (2022). Aerogels as alternatives for thermal insulation in buildings—A comparative teeny review. Mater. Today: Proc..

[27] Chen Y., Klima K., Brouwers H., Yu Q. (2022). Effect of silica aerogel on thermal insulation and acoustic absorption of geopolymer foam composites: The role of aerogel particle size. Compos. Part B: Eng..

[28] Yin Y., Song Y., Chen W., Yan Y., Wang X., Hu J., Zhao B., Ren S. (2022). Thermal environment analysis of enclosed dome with double-layered PTFE fabric roof integrated with aerogel-glass wool insulation mats: On-site test and numerical simulation. Energy Build..

[29] Song Z., Zhao Y., Yuan M., Huang L., Yuan M., Cui S. (2022). Thermal Insulation and Moisture Resistance of High-Performance Silicon Aerogel Composite Foam Ceramic and Foam Glass. Adv. Eng. Mater..

[30] Ibrahim M., Biwole P.H., Achard P., Wurtz E., Ansart G. (2015). Building envelope with a new aerogel-based insulating rendering: Experimental and numerical study, cost analysis, and thickness optimization. Appl. Energy.

[31] Elshazli M.T., Mudaqiq M., Xing T., Ibrahim A., Johnson B., Yuan J. (2021). Experimental study of using Aerogel insulation for residential buildings. Adv. Build. Energy Res..

[32] Sakiyama N., Frick J., Stipetic M., Oertel T., Garrecht H. (2021). Hygrothermal performance of a new aerogel-based insulating render through weathering: Impact on building energy efficiency. Build. Environ..

[33] National Centers for Environmental Information Climate Data Online Search. https://www.ncdc.noaa.gov/cdo-web/search.

[34] ANSYS (2018). Fluent User’s Guide, Release V19.0.

[35] Berardi U. (2015). Development of glazing systems with silica aerogel. Energy Procedia.

